# Sleep, Physical Activity, and Executive Functions in Students: A Narrative Review

**DOI:** 10.3390/clockssleep7030047

**Published:** 2025-09-04

**Authors:** Giulia Belluardo, Debora Meneo, Silvia Cerolini, Chiara Baglioni, Paola De Bartolo

**Affiliations:** 1 Department of Human Sciences, Guglielmo Marconi University, Via Plinio 44, 00193 Rome, Italy; d.meneo@unimarconi.it (D.M.); s.cerolini@unimarconi.it (S.C.); c.baglioni@unimarconi.it (C.B.); 2 Department of Psychiatry and Psychotherapy, Faculty of Medicine, Medical Center, University of Freiburg, 79104 Freiburg, Germany; 3 Laboratory of Experimental Neurophysiology, IRCCS Santa Lucia Foundation, Via del Fosso di Fiorano 64, 00143 Rome, Italy

**Keywords:** executive functions, sleep, physical activity, students, cognitive well-being

## Abstract

The school and university periods represent a critical phase in individuals’ cognitive, emotional, and behavioural development. Numerous lifestyle factors can influence executive functions and high-level cognitive processes crucial for learning and behavioural adaptation. Sleep and physical activity are two variables that influence executive functions and that could be modified through behavioural interventions. Numerous scientific studies suggest that adequate sleep quality and duration are linked to improved cognitive performance. Similarly, regular physical exercise correlates with neurocognitive benefits. However, these two aspects of lifestyle are often compromised in students, resulting in attention difficulties, reduced working memory, and difficulty in inhibitory control, all aspects of non-optimal executive functioning. Even though the scientific literature separately explores “sleep and executive functions” and “physical activity and executive functions”, few studies have integrated the two factors to assess their combined effect on executive functioning, particularly within the student population. The present narrative review aims to outline an integrated theoretical framework of existing scientific literature and to identify any knowledge gaps that may guide future research. It could provide relevant insights for designing preventive or promotional interventions to enhance students’ cognitive performance and mental well-being.

## 1. Introduction

Over the past decade, there has been growing attention to the cognitive well-being of students in both primary and higher education. Extensive research has explored the role of cognitive functions in academic performance and mental health among students. It has been demonstrated that cognitive functioning can predict academic success and help explain individual differences at both the school and university levels; conversely, poor cognitive performance is associated with psychological distress [1,2]. Among the various cognitive domains considered, executive functions (EFs) play a crucial role in the student population. EFs are higher-order neurocognitive processes that control thoughts and behaviours aimed at achieving a goal or objective [3]. The school and university periods represent critical phases in behavioural, cognitive, and emotional development, so good executive functioning becomes crucial in these life stages, where continuous integration of skills is required to achieve specific outcomes. Furthermore, EFs and academic performance can predict personal satisfaction and quality of life among students [4,5].

Extensive research indicates that many modifiable lifestyle factors can significantly influence the development of cognitive functions, with a healthy lifestyle acting as a key predictor of academic performance [6,7,8,9,10,11,12]. Among the potentially modifiable lifestyle factors impacting cognition are sleep and physical activity (PA) [6]. Sleep affects memory, learning, and the acquisition of new skills, all of which are essential for good academic performance [13]. PA improves cognitive performance, including processing speed, memory, and EFs, playing a significant role in students’ academic pathways [14,15,16,17]. Furthermore, the two lifestyle factors appear related: PA enhances sleep quality, and good sleep positively impacts PA in student populations [14,15,16].

These premises explain the emergence in the literature of numerous studies on students’ EFs and their relationship with sleep and physical activity in the student population. This narrative review aims to critically examine the existing evidence on the relationships between sleep, physical activity, and executive function among students, analysing for the first time experimental studies that have investigated the individual or combined impact of sleep and physical activity on the executive functioning of students in various age groups. Given the diversity of ways in which these variables are conceptualised, operationalised, and studied, often in a fragmented manner, using different experimental paradigms and samples of various age groups, and given the absence of both narrative and systematic reviews on this specific topic in the literature that include different age groups, this review aims to develop an innovative integrated theoretical framework that allows for a more coherent interpretation of existing findings. The goal is also to identify commonalities, inconsistencies, and gaps in the current literature, thereby outlining future directions for research on the combined role of sleep and physical activity in influencing cognitive functions.

## 2. Materials and Methods

### 2.1. Search Strategy

For this narrative review, relevant studies were identified through searches of the PubMed and Scopus databases for articles published before 31 January, with no time limit (Table S1). The search combined the following terms: “Executive functions” or “Working memory” or “Cognitive flexibility” or “Inhibition”, “Physical activity”, “Sleep”, “Students”. The same queries were applied across all databases.

### 2.2. Inclusion and Exclusion Criteria

Studies meeting the following criteria were included in the review:

1. The study population included students of different age groups (primary school, middle school, high school, and university);
2. The variables taken into consideration were executive functions (working memory, cognitive flexibility, and inhibition), sleep (sleep quality and, sleep duration), and physical activity (acute or, chronic;, aerobic or, anaerobic;, high, medium, or low intensity).
3. The articles were published in English.

Studies were excluded if they met any of the following criteria:

1. Reviews or meta-analyses;
2. Conference papers or editorials;
3. Duplicated studies;
4. Animal studies.

There were no restrictions on the year of publication for the studies considered.

### 2.3. Study Selection

After removing duplicates, all articles were evaluated and selected based on the title, abstract, and full text. The evaluation process was conducted using Microsoft Excel (Microsoft Corp., Redmond, WA, USA).

## 3. Results

**Figure 1:**
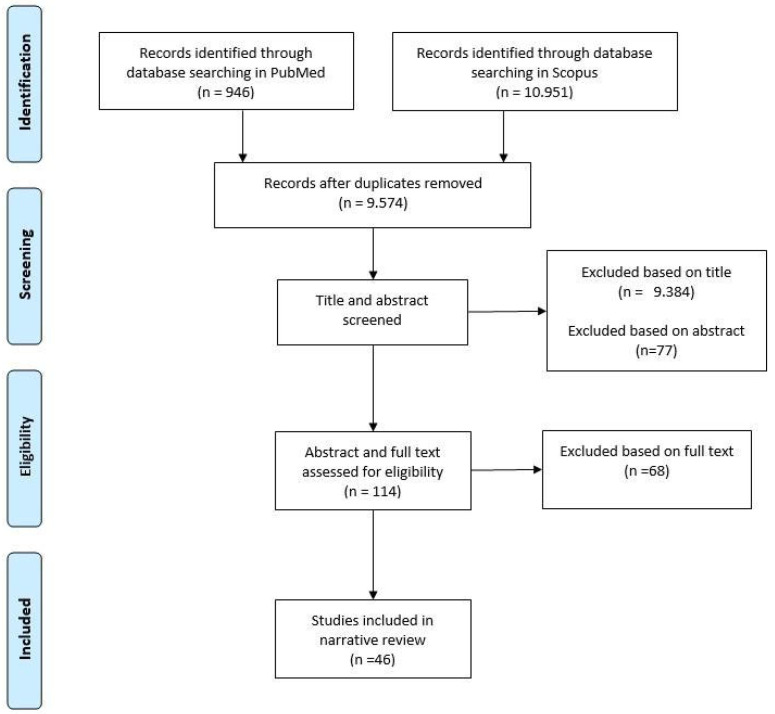
Search and selection of eligible articles.

The study selection process led to the identification of a total of 46 studies (Figure 1) that met the inclusion criteria. A total of 22 articles examined executive functions and sleep in students, of which 13 focused on sleep duration and 9 on sleep quality. A total of 20 articles examined executive functioning and physical activity. A total of 20 articles examined executive functions and physical activity in students, of which 6 examined the effects of aerobic activity and 3 examined the effects of anaerobic activity on EFs. In terms of frequency, 7 referred to chronic PA and 9 to acute PA; in terms of intensity, 2 referred to low intensity, 11 to moderate intensity, and 11 to high intensity. Of the 46 articles, 5 were about primary school students, 6 about middle school students, 7 about high school students, and 28 about university students. The 46 studies are described in the following sections: 5. Sleep and executive functions in the student populations; 7. Physical activity and executive functions in student populations; and 8. Sleep, physical activity, and executive functions in student populations.

## 4. Executive Functions

Executive functions (EFs) are a set of higher-order cognitive abilities that allow individuals to control their behaviour consciously [3,18]. Three core components that comprise the collection of cognitive processes often grouped as EFs are working memory, cognitive flexibility, and inhibitory control. Although these components operate differently, they share a common goal: the allocation of attention and regulation of behaviour to achieve an adaptive outcome [19].

Working Memory (WM) is a central aspect of EF and refers to the ability to temporarily store and manipulate information to complete complex cognitive tasks [20,21,22]. According to this definition, WM is a process that involves the active manipulation of information temporarily acquired. The development of WM begins in the early months of life. Around 12 months, a decrease in the activation of areas related to WM occurs, likely due to processes of functional specialisation. A notable improvement in WM capabilities takes place during early childhood (ages 4–7), with WM abilities subsequently increasing steadily during the years and stabilising in early adolescence (ages 14–15) [22,23]. WM is strongly correlated with specific academic performance among primary, secondary, and university students; furthermore, it appears to possess a predictive value for academic performance that is stronger than that of intelligence [24,25,26,27].

Cognitive flexibility (CF) refers to the ability to adapt one’s thoughts and behaviours in response to changes in context or specific task demands [28]. CF enables a shift from one strategy to another as necessary, thereby modifying one’s approach in response to feedback and errors. It develops rapidly during preschool age and consistently increases until adolescence, reflecting the growth of neural networks in the prefrontal cortex [29]. Students with high CF achieve better results in learning probabilistic rules and, more generally, in their academic performance [30,31].

Inhibitory Control (IC) is the ability to suppress automatic responses or distractions that may interfere with the execution of relevant tasks [32,33]. The developmental trajectory of IC begins towards the end of the first year of life, with significant improvements evident during the early childhood and preschool years. It increases at a more consistent rate during middle childhood and reaches adult levels during adolescence [22]. In an academic context, IC serves to maintain attention and avoid incorrect responses. In this regard, various studies have documented a negative correlation between the degree of IC and academic performance among students in early childhood, adolescence, and emerging adults [33,34,35,36].

The neuroanatomical substrate of EFs includes various brain areas, primarily located in the frontal lobes. The key regions involved are the Dorsolateral Prefrontal Cortex (dlPFC), responsible for working memory, goal-directed attention, task switching, planning, problem-solving, and seeking novelty; the Ventrolateral Prefrontal Cortex (vlPFC), which plays a role in IC, response selection, and monitoring; the Medial Prefrontal Cortex (mPFC), involved in self-awareness, motivation, updating goal-directed behaviour, and emotional regulation; the Orbitofrontal Cortex (oFC), associated with IC and emotional and social reasoning; and the Fronto-Aslant Tract (FAT), involved in action control tasks, planning, timing, and coordinating sequential motor movements, as well as resolving competition between potential voluntary movements. These areas work together to support the complex processes involved in executive functioning [37,38,39,40].

## 5. Sleep

Sleep is a necessary function for life, shared by all animals [41]. It is a resource-intensive activity that performs a range of vital functions, including immune responses, development, and energy balance [40,41]. A two-process model governs sleep–wake rhythms: Process S, or the homeostatic process, promotes sleep based on the length of prior wake periods, reflected in the power density of slow-wave activity during non-rapid eye movement (NREM) sleep; Process C, or the circadian process, maintains wakefulness, independent of prior sleep and wake periods, and reflects the endogenous circadian system regulating the sleep–wake cycle [42,43,44]. The neuroanatomical area that serves as the pacemaker for this system is the suprachiasmatic nucleus, synchronised with the external light–dark cycle [45]. The sleep period consists of two main phases: Rapid Eye Movement (REM), characterised by rapid eye movements, muscle atonia, rapid low-voltage theta waves, and fluctuations in heart and respiratory rates; NREM, which includes Stage 1, Stage 2, and slow-wave sleep (SWS), characterised by reduced neural activity and low-frequency, high-amplitude delta waves [46,47]. The duration, quality, and architecture of sleep change throughout life, especially during the first five years and in adolescence. Infants, unlike adults, spend up to 80% of their day sleeping, while most toddlers and preschoolers sleep for more than half of the day. This variation in sleep patterns is crucial for their overall development. The daily sleep duration of infants, generally ranging from 14 to 20 h, is halved before adolescence. In the early months of life, sleep is distributed across multiple periods with numerous naps; however, by ages 5 to 7, it consolidates into a single period, and naps significantly decrease [48,49,50].

### Sleep and Cognitive Development

Factors such as sleep duration, quality, chronotype, and architecture influence cognitive development and the functioning of cognitive abilities throughout life [51,52,53]. Many studies support a facilitating or direct physiological role of sleep in various cognitive domains, including executive functioning. During development, the most important facilitators of cognitive processing seem to be sleep spindles and slow waves [49,52]. Individual differences in sleep duration and fragmentation also play a role in cognitive development. Pisch et al. (2019) demonstrated that children who spend less time awake at night during early childhood perform better on WM tasks [54]. A plausible physiological explanation for this improvement is the increased duration of deep sleep (SWS) in children.

Research shows that the EFs of preschool children are also supported by sleep: children with greater sleep efficiency (i.e., a higher ratio of time spent sleeping over time in bed) performed better on executive tasks, and sleep quality is a predictor of WM, CF, and IC [55]. Similarly, in primary school children, sleep is correlated with performance on WM, CF, and IC tasks. Children aged 6 to 12 show improvement in executive tasks, such as planning and problem-solving, only after a period of sleep, not after an equivalent period of wakefulness [56,57].

Sleep continues to impact cognitive development during adolescence as well. During this time, bedtimes are often delayed, school schedules determine wake times, and total sleep time decreases, despite the “need” for sleep remaining unchanged since school age [58]. During adolescence, the main physiological changes in sleep include a reduction in slow-wave sleep (SWS), an increase in Stage 2 sleep, and a delay in the circadian rhythm, which is reflected in sleep onset times. Various studies examined the relationships between the neurophysiological characteristics of sleep during adolescence and cognitive functioning. Some of these describe a positive correlation among adolescents between intelligence quotient, EFs, and the characteristics of sleep spindles in terms of power, amplitude, and intensity, and an association between sleep duration and the functional connectivity of attentional, executive, mnestic, and sensory networks during early adolescence [58,59,60]. Circadian rhythms, and more specifically chronotype, also influence cognition during adolescence. Circadian rhythms vary considerably from person to person, and individuals can be classified according to their circadian type (also known as chronotype or circadian preference) as morning types (M types), intermediate types (N types/I types), or evening types (E types), depending on their sleep habits and preferred activity times [61]. Evidence regarding the impact of chronotype on adolescent intelligence suggests age as an important moderator in the chronotype–intelligence relationship. In fact, it would appear that the relationship between M-type and intelligence shifts from positive to negative with advancing age and reaches significant levels only in young adults over 25, with higher intelligence for E-types [62]. Executive functions are also influenced by chronotype, with studies suggesting the presence of a “synchrony” effect whereby executive performance tends to be better at times of the day that coincide with the peak activation of one’s circadian rhythm [53]. The literature also demonstrates that manipulating sleep can modulate cognitive functioning during adolescence. Studies that employ brief sleep disturbances, through daytime naps, sleep restriction over several days, or total sleep deprivation (SD), showed that the restorative benefits of sleep or the detrimental impact of sleep loss is reflected in performance on attention tasks, EFs, emotional regulation, and learning and memory [63,64,65,66]. Although these studies do not directly measure the long-term consequences of altered sleep, they provide important data on the impact of short-term night-time sleep loss and daytime napping on cognition during adolescence.

The emerging adult age group, which spans from 18 to 29 years, is characterised by further changes in sleep duration and quality. Emerging adults are described as a social group facing numerous challenges related to the transition to adulthood, such as increased responsibilities, the ability to make autonomous decisions, and, for some, attending university. These factors contribute to uncertainty, elevated stress levels, and sleep disturbances [67]. During the transition to university, there is a tendency for emerging adults to delay their night-time sleep by about two hours, resulting in a reduction in overall sleep duration. An extensive study conducted on emerging adults reported an average night-time sleep duration of less than 7 h [68]. Accompanying this reduction in sleep hours are irregular sleep–wake cycles and a shift in circadian rhythm towards an evening-oriented preference, which begins during adolescence and peaks between the ages of 17 and 21. This tendency often reverses with a return to morning-oriented sleep–wake rhythms during adulthood [69,70,71]. Silva and colleagues (2010) found that irregular sleep–wake cycles have a greater impact on cognitive ability in young adults compared to older adults [72]. This suggests that changes associated with emerging adulthood significantly affect cognition. In this life stage, insufficient sleep duration and symptoms of insomnia are related to impairments in attention performance and WM [73,74,75].

From all this evidence, it is clear that sleep plays a crucial role in cognitive functioning throughout development and should be considered when creating pathways to improve cognitive performance.

## 6. Sleep and Executive Functions in Student Populations

The student population has a prevalence of sleep disorders of around 30%. It is widely recognised that sleep is closely linked to WM, CF, and IC, and that sleep disorders can affect academic performance [76]. The most common sleep disorders among students are narcolepsy, restless legs syndrome, insomnia, and obstructive sleep apnoea. These disorders clearly alter the duration and quality of sleep [77]. Studies on students have shown that longer, higher-quality, and more regular sleep is associated with better academic performance. Conversely, a week of partial SD can impair EFs, with effects that persist even after two nights of recovery. It is important to recognise that the effects of different sleep durations and quality may vary during development, thus affecting different groups of students and their academic performance at school or university in various ways [78,79]. A comprehensive understanding of the impact of sleep duration and quality on students’ executive functions is essential to develop pathways that can enhance their quality of life during development and support satisfactory academic achievement.

### 6.1. Effects of Sleep Duration on Executive Functions

Sleep duration plays a crucial role in maintaining EFs. Studies show that sleeping between 6 and 8 h a day is associated with greater activity in the oFC, hippocampus, and prefrontal cortex (PFC), and higher scores on EFs. Conversely, SD is associated with reduced brain volume in specific regions and lower cognitive performance [80]. Such evidence is lacking in the paediatric population but becomes increasingly available as development progresses, leading to a multitude of studies in the university population. Among the very few studies that have investigated the relationship between sleep duration and EFs in school-aged children is the one conducted by Warren et al. (2016) [81]. The research revealed that a longer night-time sleep duration in fourth-grade children is linked to improved executive performance, especially in IC tasks.

Adolescence, unlike late childhood, is a stage that has been more thoroughly researched. D’Angiulli et al. (2023), for instance, investigated the effect of sleep duration and social jet lag (SJL, i.e., a mismatch between social and biological schedules) on IC, without altering sleep hours. The results indicate that reduced sleep duration, caused by adolescents’ tendency to sleep less, impairs IC, as shown by both behavioural measures—reporting more errors and slower reactions in Stroop tasks—and neurophysiological indicators, such as less-efficient event-related potentials (ERPs) and increased delta EEG activity. The phenomenon appears to be more pronounced on days with high SJL and following an ecological accumulation of SD [82]. Conversely, the study by Zhang et al. (2024) examined the effects of acute 24 h SD on the CF of adolescent students. The accuracy in the SD group was lower than that in the control group, indicating a negative impact of SD on CF. At the neurophysiological level, the study observed that ERP components, such as N1, P2, N2, P3, and LNC, exhibit significant differences between groups, indicating that SD affects both behavioural performance and the underlying neurophysiological processes [83]. Another important aspect highlighted by Cohen-Zion & Shiloh (2017), relating to the adolescent student population, is the consequences of SD, such as daytime sleepiness, and the impact these have on EF. It has been demonstrated that sleepiness caused by inadequate sleep duration has an immediate and significant impact on EF, surpassing the direct effect of the number of hours slept [84]. In summary, reduced sleep duration significantly impacts the EFs of adolescent students both directly and indirectly.

As mentioned above, most studies have focused on examining the relationship between sleep duration and EFs in emerging adult students, distinguishing between the effects of total, partial, acute, and chronic SD on EFs. Among these, the study conducted by Pace-Schott et al. (2014) examined the effects of acute total sleep deprivation (TSD) on EFs in university students in a naturalistic setting. Contrary to initial expectations, the research found that a single night of TSD did not cause significant deficits in EF performance compared to the control group. The authors suggest possible explanations for these findings, including the resilience of young adult students to short episodes of deprivation, or the fact that both groups already had some “chronic sleep restriction” that may have masked the effects of acute deprivation [85]. A few years later, Yeung et al. (2018) investigated the effect of acute partial SD on WM and frontal activation in a student population of young adults using near-infrared spectroscopy. Again, participants with insufficient sleep (IS) performed similarly on WM tasks compared to participants with sufficient sleep (SS). However, at the neurophysiological level, SS students showed clear bilateral activation of the prefrontal cortex during the WM task. In contrast, the IS group showed no significant changes during the task, indicating an absence of frontal activation in response to the WM load. Therefore, partial SD of two hours does not cause significant changes in cognitive performance, but it alters the activation of the prefrontal cortex [86]. It is interesting to note that, upon increasing SD to 36 h, Peng et al. (2020) found a significant deterioration in some aspects of executive performance, particularly WM. The study, through the analysis of ERPs, also found a decrease in the amplitude and an increase in the latency of the N2 and P3 components. These alterations reflect a reduction in attentional resources and difficulty in suppressing responses and, therefore, in IC [87]. The results of a study conducted by Cerolini et al. (2020) on university students suggest that partial SD can selectively impair IC in people with binge eating symptoms, whereas this does not occur in individuals without disordered eating behaviours. Therefore, certain conditions, such as eating disorders, can affect the sleep–EF relationship [88]. Jin et al. (2015), through a study with TSD (up to 36 h) on university students, also found a dose-dependent effect, whereby, the more SD progresses, the more notable the impairment in IC [89]. SD, therefore, seems to have a cumulative nature in its negative effects. This evidence was confirmed by a study by Del Angel et al. (2015), examining the effects of 5 days of partial SD on the phonological and visuospatial components of WM in undergraduate students. The results revealed that 5 days of SD at four hours per night negatively affect both components of WM. In particular, the phonological component of WM decreased on the fifth day of SD and did not fully recover after a night of recovery sleep [90]. From this evidence, it can be said that the progressive alteration of EFs, caused by a chronic increase in SD, reflects a cumulative effect of sleep towards negative outcomes. However, Ballesio et al. (2018) also demonstrated that individuals who report symptoms of chronic insomnia, after one night of partial sleep deprivation, do not report any change in executive performance. Several factors may explain these results. The heightened level of cognitive arousal usually shown in CI may have paradoxically facilitated their executive performance [91]. It has been reported that CI tends to mobilise extra cognitive resources to compensate for their deficits [92], which may result in habitual efficient performance.

The negative effects of insufficient sleep duration can be mitigated by recovery sleep. In this regard, Jin et al. (2015) found that, after 36 h of TSD, 8 h of sleep improves the latency of the P3 component and the latency of the N2 component, indicating a recovery of IC [89]. However, the recovery is only partial because neither the neurophysiological indices nor the cognitive performance fully return to the original levels prior to SD. Conversely, if SD impairs EFs, then a nap in addition to the usual hours of night-time sleep can enhance WM and IC performance in university students. Lau et al. (2015) assessed the impact of a 90 min nap on WM and observed a notable increase in WM accuracy, alongside a positive correlation between REM sleep duration and WM. REM sleep plays a key role in the functionality of the PFC, an area that is fundamental for WM. During REM sleep, PFC activation and connectivity increase, suggesting a possible mechanism linking sleep and PFC that underlies the observed improvement. Furthermore, the benefit of napping was shown to be independent of the quality of previous night-time sleep [93]. These data indicate that, even in conditions of mild chronic SD, which is very common in young university students, napping may offer compensatory benefits for EFs.

**Figure 2:**
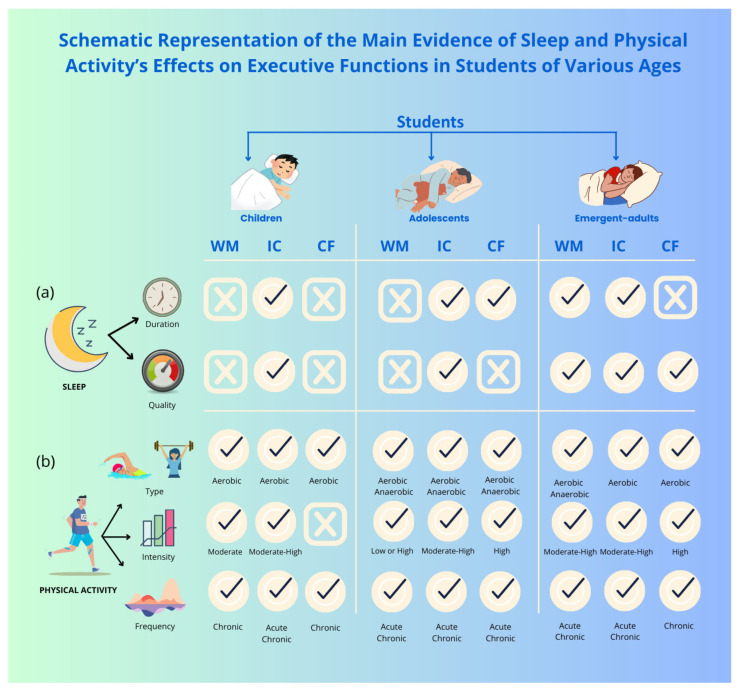
Schematic representation of the main evidence of sleep and physical activity’s effects on executive functions in students of various ages. The figure summarises the key evidence from the revised literature separately examining the impact of (**a**) “Sleep” (Duration, Quality) and (**b**) “Physical Activity” (Type, Intensity, Frequency) on “Executive Functions” (Working Memory, WM; Inhibitory Control, IC; Cognitive Flexibility, CF) in students of different ages (Children, Adolescents, Emergent-Adults). Hooks show evidence present; crosses show evidence absent.

The upper part of Figure 2 presents a schematic representation of the main findings described in this paragraph.

### 6.2. Effects of Sleep Quality on Executive Functions

Unlike sleep duration, Sleep Quality (SQ) is a composite concept. SQ is defined as an individual’s satisfaction with various aspects of their sleep experience. It is often conceptualised as based on four sub-dimensions, such as the efficiency, latency, and duration of sleep and wakefulness after falling asleep, as well as on antecedent factors including physiological aspects (age, circadian rhythm, amount of REM or NREM sleep), psychological factors (e.g., stress, anxiety, depression), environmental factors (lighting, temperature, etc.), and social and family factors [94]. SQ within the student population is not always optimal, and there are age groups where it is considerably lower. A cross-sectional study involving 9.392 students from primary, middle, vocational, high school, and university levels reported a progressive decline in SQ, with 7.5%, 19.2%, 28.6%, 41.9%, and 28.5%, respectively, exhibiting poor scores in SQ. High school students reported the highest prevalence of inadequate sleep duration, accompanied by daytime dysfunction. Elementary school students showed the highest prevalence of low sleep efficiency, and university students showed the highest prevalence of sleeping pill use. Generally, SQ is positively associated with school term, grades, and academic pressure [95]. How SQ impacts EFs is widely studied in the literature; however, not all age groups are covered with the same number of research studies. Primary school students have been little investigated, and one of the very few studies on SQ and EFs concerns a population of 6- to 10-year-old students. Higgins (2020) found that daytime sleepiness predicts lower IC; however, the absence of a scale that offers an overall SQ value prevents us from determining how much the different dimensions of SQ influence EFs in this group of students [96].

The adolescent student population is also under-researched and, in some cases, lacks standardised questionnaires that evaluate SQ globally. A study by Anderson et al. (2009) offers data similar to those previously discussed for school-age children. SQ was evaluated through a combination of various parameters, such as sleepiness, sleep duration, and efficiency, using questionnaires, actigraphy, and polysomnography [97]. Among the many parameters used to assess SQ, sleepiness is the one most strongly linked to EFs in adolescent students. A more recent study by Ouellet et al. (2024), which used a retrospective questionnaire specifically designed to assess sleep quality, confirmed that low QS is associated with poorer executive performance and is strongly linked to IC [98].

The evidence increases for the university population, where standardised SQ questionnaires, which are widely used in the literature and suitable for comparing populations, such as the Pittsburgh Sleep Quality Index (PSQI), have been employed. Several studies, like those by Gong et al. (2024), Parrilla et al. (2024), and Almarzouki et al. (2022), show that poor SQ in university populations is linked to poorer WM performance [99,100,101]. Gong et al. (2024) also demonstrate that SQ is a predictor of visual WM [99]. However, it is not only WM that is affected by SQ in the university population. Using the PSQI, Conner (2015) demonstrates that low SQ in university students correlates with numerous executive skills, including IC [102]. Abbas et al. (2020) showed that IC is positively correlated with a significant aspect of SQ, the amount of REM sleep. CF is less investigated in this field of study [103]. It has been found that QS also influences it in the university population. A study by Chen et al. (2024) showed that low SQ, assessed using the PSQI, is associated with significant symptoms of insomnia, leading to an alteration of CF abilities and a reduction in the amplitudes of the P2, N2, and P3 components detected using ERPs. These components are associated with selective attention allocation (P2), IC (N2), and WM (P3), respectively [104].

Overall, the literature in this field requires more research on school-age children and adolescents. Moreover, the relationship between CF and SQ is among the least explored areas across all student categories, and additional data could offer a more comprehensive understanding of the impact of SQ on EFs.

The lower part of Figure 2 presents a schematic representation of the main findings described in this paragraph.

## 7. Physical Activity

Physical activity refers to any form of body movement caused by muscle contractions [105]. Therefore, PA is not limited to sports but also includes activities such as walking, cycling, light gymnastics, and playing. The compendium of PA is useful for estimating the metabolic intensity of the activity in relation to a resting phase in terms of metabolic equivalent tasks (METs). The METs are used to categorise activities, which can be sedentary or inactive, moderate-intensity activities, or vigorous activities [106]. Among low-intensity sedentary activities, examples include watching television, doing office work, and slow walking. Moderate-intensity activities include leisurely cycling or gardening. High-intensity activities include sports such as cycling, jogging, and running.

PA is categorised into two types: aerobic and anaerobic. Aerobic exercise is generally defined as any activity that involves large muscle groups, can be sustained continuously, and has a rhythmic nature. The muscle groups activated by this type of activity rely on aerobic metabolism to extract energy in the form of adenosine triphosphate (ATP) from amino acids, carbohydrates, and fatty acids [107]. Aerobic activities, such as cycling, swimming, and running, can be assessed through aerobic capacity, which is the ability of the cardiorespiratory system to supply oxygen and the skeletal muscles’ ability to utilise oxygen. In contrast, anaerobic exercise is a short-duration, intense PA that derives energy from sources present in contracting muscles and is independent of inhaled oxygen as an energy source [108]. This type of exercise produces ATP via glycolysis and fermentation, resulting in the accumulation of lactic acid. Usually, anaerobic exercises, such as powerlifting or high-intensity interval training (HIIT), cause a sustained increase in lactate and metabolic acidosis, with this transition point known as the anaerobic threshold, which can be measured through frequent blood samples assessing lactate levels [109,110].

Finally, PA is also divided into acute and chronic forms. Acute PA involves a single session of exercise, while chronic PA includes repeated exercise over time, often measured in weeks and months [111].

PA has a powerful positive impact on many aspects of health. It can prevent and treat non-communicable diseases, enhance resilience against the development of mental illnesses, and mitigate cognitive decline [112,113,114,115]. Conversely, various studies have reported that physical inactivity increases the risk of mortality, leads to poorer overall health conditions and lower life expectancy, and is strongly associated with poor sleep quality [116,117,118]. Physical activity is recognised as an important determinant of cognitive and neural functioning, during both adulthood and development. Physical movement, through an increased blood flow to the brain and the release of neurochemical substances, promotes cognition. The human brain constitutes approximately 2% of the human body’s mass; however, it requires around 20% of the energy consumed. With PA, blood flow increases, and consequently, the supply of oxygen and nutrients to the brain improves, enhancing brain activity [119,120,121].

### Physical Activity and Cognitive Development

Starting from the premise that habits related to PA acquired during early childhood are moderately sustained into later childhood, many recent studies have explored PA and health. A particularly sensitive period of cognitive development, school age (5–10 years), has been the focus of numerous studies examining the influence of PA on cognition [122]. A systematic review considering both longitudinal and cross-sectional studies supports the idea that PA has a beneficial effect on cognition during the school-age years. In particular, the examined cross-sectional studies revealed that physically fit children achieve better cognitive performance compared to less-fit children. Longitudinal studies, on the other hand, suggest that higher levels of fitness or an increase in PA are predictors of improved cognitive performance. Specifically, the evidence indicates that individual sessions of short-term PA selectively enhance cognitive performance in terms of speed and accuracy. However, in-depth analyses reveal that regular or chronic PA impacts children’s cognitive performance on specific mental tasks and can also modify brain structure and functions. Furthermore, evidence regarding the dose–response relationship revealed that the attendance rate at PA sessions is associated with changes in neural indices of attention allocation, faster cognitive processing speeds, and better behavioural performance during executive tasks [123]. Moreover, the cognitive performance of school-aged children appears to improve with the frequency of PA sessions: more sessions enhance cognitive performance. The effects of the type of PA and the duration of PA sessions on this population remain to be explored [124].

Physical activity has a notable impact on cognition during adolescence as well. This stage of life is characterised by significant brain plasticity, providing numerous opportunities for developing cognitive abilities [125]. Specifically, some ecological evidence suggests that moderate-intensity PA, performed consistently during adolescence, is associated with improved cognitive performance. Conversely, high-intensity and high-frequency PA may impair adolescents’ cognitive performance [126]. It is not surprising that PA levels tend to decline during adolescence [127]. An explanation for this evidence may lie in how adolescents allocate their daily time. Very frequent PA could detract from time spent on activities that equally influence cognitive development, such as school assignments, personal reading, and other cognitively stimulating educational activities. Furthermore, some studies reported that aerobic PA in adolescents is associated with faster reaction times and better IC, unlike high-intensity anaerobic PA patterns [128]. Aerobic PA during adolescence is also positively correlated with spatial learning and associated with visual WM [129,130].

Emerging adulthood (ages 18–29) occurs during a phase of brain and cognitive development, as both grey and white matter continue to develop until around age 30. More pronounced changes are seen in the PFC [130,131,132,133,134]. In this context, a recent meta-analysis examined the effects of both acute and chronic PA on the cognitive functioning of emerging adults. It is generally found that acute PA has a moderate impact on cognitive outcomes; notably, a significant moderate effect was observed specifically on processing speed, attention, and EFs. Further analysis of the EFs revealed that acute PA has a moderately substantial impact only on IC, with no significant effects on cognitive CF or WM. On the other hand, chronic PA produced moderate effects on processing speed, language, and EFs. The distinction among the EFs has shown a small impact of chronic PA on CF and a significant effect on WM [135]. Furthermore, other evidence reveals that aerobic PA is associated with the cognitive functioning of emerging adults. More specifically, aerobic capacity appears to be linked to improvements in WM and attention [136]. Regarding the effect of anaerobic PA in emerging adults, the literature is lacking in studies.

In general, these data strongly support the role of PA in cognitive development, laying the groundwork for further research in the field.

## 8. Physical Activity and Executive Functions in Student Populations

As mentioned above, PA has various forms that have specific effects on students’ executive functioning. Exploring which of these variations—aerobic or anaerobic; acute or chronic; and high, moderate, or low intensity—are most effective in positively influencing EFs and which might burden cognitive performance in different student groups is a key goal of many studies.

### 8.1. Effect of Aerobic and Anaerobic Activity on Executive Functions

The effect of aerobic activity on the EFs of the student population has been investigated more extensively than the effect produced by anaerobic activity. Once again, evidence from primary school students is limited. Ren et al. (2024) investigated the impact of 16 weeks of aerobic activity on WM, IC, and CF in primary school children. They demonstrated that aerobic PA leads to a significant improvement in WM, IC, and CF, and that these improvements are even greater when aerobic activity is combined with cognitive activities. These data support including programs that combine movement and cognitive stimulation in schools to promote the optimal development of children’s EFs [137]. Also, during adolescence, aerobic PA has been shown to significantly improve EFs.

An interesting finding of Erwin et al. (2024) concerns the comparative effectiveness of aerobic and anaerobic PA in the adolescent student population. The results showed that aerobic PA produces positive effects on WM, unlike anaerobic PA, which improves reaction times [138]. Hu et al. (2024) investigated the effects of aerobic exercise combined with anaerobic resistance training on EFs in obese (OA) and normal-weight (NWA) adolescent students. Before the intervention, the OA students had significantly lower IC and CF levels than the NWA students; after the intervention, the OA students reported a significant improvement in both IC and CF, aligning with the performance of the NWA students. There were no differences in WM before the intervention between the two groups of students, but after the intervention, the OA students reported significant improvements in WM [139]. In sum, combining aerobic and anaerobic exercise can also enhance EFs in obese adolescents.

Many more studies have investigated the effect of aerobic and anaerobic PA on EFs in university students. Several studies, including those by Ludyga et al. (2018) and Fan et al. (2021), agree that aerobic PA in university students can significantly improve IC and CF, especially when exercise is combined with cognitive training [140,141]. However, the effects of aerobic exercise on WM are less consistent. Li et al. (2014), using functional magnetic resonance imaging, showed that a single session of aerobic exercise leads to greater activation of the PFC, right lingual gyrus, and left fusiform gyrus, areas involved in WM and complex visual processes. However, these changes did not translate into significant improvements in WM performance [142]. In contrast, Martínez-Díaz et al. (2020) demonstrated that a single session of anaerobic exercise, based on a High-Intensity Interval Training (HIIT) protocol, significantly improves verbal WM performance immediately after the PA session and up to 30 min later [143].

Overall, this evidence indicates the use of aerobic and, in the university setting, anaerobic practices, ideally combined with cognitive training, to improve their positive impact on EFs.

The upper part of Figure 1 presents a schematic representation of the main findings described in this paragraph.

### 8.2. Effect of Frequency and Intensity of Exercise on Executive Functions

The type of exercise in terms of the frequency and intensity adopted leads to differences in the effects of PA on students’ EFs. Liu et al. (2024), for example, found that, in primary school populations, PA with high frequency and moderate-high intensity can improve CI. On the other hand, Ren et al. (2024) demonstrated that a chronic PA intervention of moderate intensity (16 weeks) on primary school students can significantly improve WM and that, if chronic PA is combined with cognitive training, IC and CF also improve [137,144].

Studies of the same type also focused on adolescent students. Peruyero et al. (2017) found that acute moderate-to-high-intensity PA is more effective than low-intensity PA in improving IC performance, demonstrating that not only the frequency but also the intensity of PA is a key element in this field of study. In fact, the effect of different intensities of PA is not the same for all EFs [145]. In this regard, Ludyga et al. (2018) demonstrated that adolescents report improvements in IC after acute PA of moderate intensity; however, this frequency and intensity would not lead to any significant improvement in WM [140]. The study by Budde et al. (2009) highlights the relationship between WM and varying intensities of PA. It shows that high-intensity PA does not enhance WM performance but instead results in an increase in salivary testosterone levels, which correlates negatively with WM. In adolescents, acute low-intensity PA appears to improve WM [146]. Unlike WM, CF seems to improve following high-intensity acute PA, and Berse et al. (2015) found that this improvement is predicted by polymorphisms linked to the dopamine transporter and D2 receptor, suggesting the involvement of the frontostriatal dopaminergic system as a mediator. From these data, it can be seen that, in adolescence, IC and CF benefit from moderate-to-high-intensity acute PA interventions [147]. In contrast, WM benefits from low-to-moderate-intensity acute PA interventions. It is interesting to note that the effects on CF in adolescent students remain virtually the same if PA is performed chronically, while there are conflicting results on the effect of chronic physical activity on IC [148,149]. WM, on the other hand, relates to chronic PA differently from acute PA. In particular, Jeon et al. (2017) found that moderate chronic PA leads to an improvement in WM only after the 14th week, unlike the improvements in IC and CF, which are detectable after 12 weeks, indicating that longer periods are required to see benefits in WM. A further finding from this study is that, unlike the data obtained on acute PA, WM in adolescent students can also improve following high-intensity PA, provided that it is chronic and lasts for at least 12 weeks [150].

Once again, the university population is the most investigated. Unlike that for primary school and adolescent students, the evidence is not homogeneous. While many studies [151,152,153] showed that moderate- or high-intensity PA is more beneficial to IC than low-intensity acute PA, another study claimed the exact opposite, demonstrating that low-intensity acute PA can improve IC more than moderate- or high-intensity acute PA [141]. This heterogeneity may depend on the type of cognitive test used to assess IC, the baseline level of the students, and, therefore, the “ceiling effect”, as well as methodological and physiological factors. In general, there is more evidence in favour of a beneficial effect of moderate-to-high-intensity acute PA on IC. Notably, in university students, WM seems to behave in the same way as IC, unlike in previous age groups. Martínez-Diaz et al. (2023), for example, found a lasting improvement in WM up to 30 min after high-intensity acute PA [154]; Ji et al. (2017) observed an improvement following acute moderate-intensity PA, and Li et al. (2014), despite not finding improvements in WM performance after acute moderate PA, detected an increase in activity in the right middle frontal gyrus, an area typically involved in executive functioning and particularly in WM tasks [142,155]. In summary, moderate-intensity PA is an optimal strategy for improving both IC and WM in university students. Positive effects have also been observed for high intensity, especially in reaction times, provided that fatigue is not excessive, as highlighted by Fan et al. (2021) [141]. Moderate and high-intensity exercise continues to produce positive effects on IC and WM, even when performed chronically. Specifically, Wang et al. (2023) demonstrated that 12 weeks of Tai Chi significantly improved WM and IC, while also leading to a significant increase in the power of θ (theta) and α (alpha) waves in the frontal cortex, as measured by EEG. These changes are associated with a more relaxed mental state, which is optimal for cognitive control, concentration, and greater CF [156]. On the other hand, another interesting study by Wang et al. (2023) investigated the effect of chronic high-intensity PA on WM, IC, and CF, distinguishing between a low dosage (once a week) and a moderate dosage (twice a week). The results indicate that a moderate dosage of chronic high-intensity PA brings greater benefits to the EFs than a low dosage [157].

In summary, both acute and chronic PA can improve students’ EFs. However, it is important to emphasise that most studies primarily assess the immediate or short-term effects of chronic PA. This methodological approach is a common limitation across the examined studies. Currently, further research involving assessments at different intervals after the intervention ends is necessary to determine whether and how long the benefits for EFs persist.

The middle and lower parts of Figure 2 present a schematic representation of the main findings described in this paragraph.

## 9. Sleep, Physical Activity, and Executive Functions in Student Populations

Although many studies in the literature have examined the relationship between sleep and EFs, and that between PA and EFs, very few have explored these three factors together in the student population.

Evidence pertaining to the adolescent students provided by Tavakoli et al. (2024) indicates a beneficial effect of chronic PA on both sleep and EFs, without investigating moderating effects [158]. Instead, Sun et al. (2022) used an ecological method to identify sleepiness as a moderator between PA and WM, finding that non-sedentary behaviours and moderate-to-high PA performed spontaneously led to less sleepiness and better performance in WM tasks [159].

There is limited data available regarding university students. A study by Li et al. (2021) confirms the data on sleep as a moderator of the relationship between PA and EFs, in particular IC. The observational study showed that the relationship between PA and IC is not purely direct but mediated by sleep quality and efficiency [160]. Therefore, according to this study, regularly engaging in PA contributes to enhancing sleep quality and efficiency; in turn, these improvements positively influence IC performance. Another interventional study, conducted by Liu et al. (2022), examined the effects of an acute aerobic PA session on students’ EFs following one night of TSD. As expected, TSD significantly impaired executive performance, particularly in IC, and significantly reduced serotonin (5-HT) levels. However, a 30 min session of aerobic PA significantly improved accuracy and reaction times on IC tasks and significantly increased serotonin levels [161]. Although limited, this evidence confirms the mediating role of sleep between PA and EFs; however, it also highlights the key role played by PA, which is able to improve executive performance even when poor sleep quantity and quality could compromise it.

**Figure 3:**
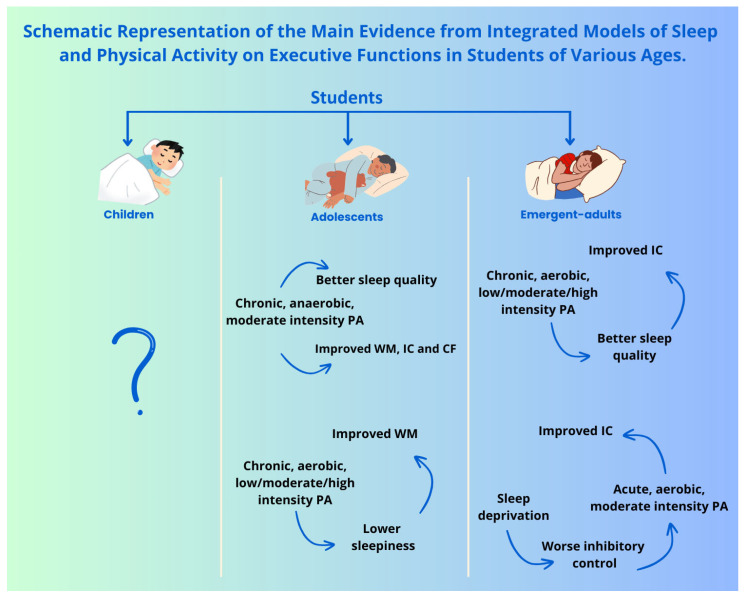
Schematic representation of the main evidence from integrated models of Sleep, Physical Activity, and Executive Functions in students of various ages. The figure summarises the key evidence from the little reviewed literature examining, in the same model, the impact of “Sleep” and “Physical Activity” on “Executive Functions” (Working Memory, WM; Inhibitory Control, IC; Cognitive Flexibility, CF) in students of different ages (Children, Adolescents, Emergent-Adults). The question mark indicates the absence of evidence in the children’s population.

These results highlight and confirm the importance of investing in PA and good sleep health together to promote students’ cognitive well-being. Further studies are needed to confirm the findings obtained and to gather data on primary school students, for whom there is currently no evidence in the literature on the combined effects of EFs, PA, and sleep. Furthermore, future studies should investigate the combined effect of sleep and PA on CF, a component of EFs that is usually less investigated than IC and WM, especially in these protocols, where it is not mentioned. Figure 3 presents a schematic representation of the main findings described in this paragraph.

## 10. Discussion

The results of this review confirm that lifestyle is a crucial determinant of cognitive development and academic performance in students [6,7,8,9,10]. In particular, sleep and PA are central modifiable factors with significant impacts on EFs, a set of higher-order cognitive abilities that allow individuals to control their behaviour consciously [13,14,15,16].

The reviewed studies highlight the critical role that sleep and PA play, both individually (Figure 1) and in combination (Figure 2), on the EFs of students in all age groups (children, adolescents, and emergent adults).

### Overall, Sleep Has a Positive Effect on All Domains of EFs

Evidence indicates that adequate sleep duration (6–8 h) is associated with better EFs. SD, both acute and chronic, demonstrates dose-dependent negative effects, including behavioural and neurophysiological changes such as increased latency and decreased amplitude of ERP components related to executive processing. The main limitation of the current literature on sleep duration is the lack of evidence for the paediatric population. The study by Warren et al. (2016), which shows a positive impact of sleep duration on IC, is an exception; further data are needed to better understand the early development of executive functions in relation to sleep duration [81]. During the adolescent stage, the data show that reduced sleep duration significantly impacts the IC and CF, both directly and indirectly [83,84]. An interesting finding for university students is that napping may offer compensatory benefits for EFs (WM and IC) even in conditions of mild chronic SD [93], which is very common in young university students.

SQ is also confirmed as a significant predictor of EFs: poor SQ is associated with poorer performance, particularly in CI and WM, especially in university students. In particular, sleepiness seems to be strongly linked to the EFs of adolescent students and to have a moderating effect between PA and WM, in line with the evidence in the literature on the relationship between sleepiness, EFs, and academic performance in adolescent and emerging adult students [84,96,97,159,162,163,164,165]. This phenomenon could be caused by a reduction in sleep duration typical of adolescence and emerging adulthood. During these phases, there is a tendency to delay bedtime, resulting in partial sleep deprivation that impacts overall sleep quality. One of the primary strengths of the examined studies is the use of standardised tools (such as the PSQI) within the university population, which permits reliable comparisons between studies and facilitates the correlation of specific aspects of sleep quality with executive domains such as WM, IC, and CF [99,100]. Neurophysiological evidence (e.g., alterations in the ERP components P2, N2, and P3) further confirms the impact of sleep quality not only on behaviour but also on the underlying cerebral processes [104]. However, significant challenges emerge. Firstly, critical knowledge gaps remain in school-aged children and adolescents. The measurement tools for SQ in these age groups are often non-standardised or fragmented, making it difficult to evaluate the overall influence of sleep quality on EFs. For example, in the study by Higgins (2020), the absence of a composite measure of SQ prevents the accurate identification of which sleep dimensions most significantly impact CI [96]. A second limitation is the poor attention to CF, which remains underexplored compared to other executive domains, diminishing the overall understanding of the impact of sleep quality on executive functions.

On the other hand, PA is a clear and powerful modulator of EFs, with significant differences depending on type (aerobic and anaerobic), frequency (acute and chronic), and intensity (low, moderate, and high).

Aerobic PA has positive effects on IC, WM, and CF in student populations, particularly when combined with cognitive stimulation. Thus, one of the strengths of recent studies is recognising the multimodal effectiveness of interventions: combining aerobic activity and cognitive training seems to enhance the benefits across various aspects of EFs, as shown by Ren et al. (2024) in children and Ludyga et al. (2018) in university students [137,140]. This approach offers important practical implications for implementing targeted educational programmes aimed at cognitive development. The efficacy of anaerobic activity appears less systematic and more controversial. While it enhances responsiveness and, according to some studies, verbal WM, it does not show the same widespread benefits for IC and CF as aerobic activity, as noted by Erwin et al. (2024) [138] and Martínez-Díaz et al. (2023) [154]. Additionally, methodological differences (intensity, duration, modality) complicate direct comparisons between exercise types.

The studies examining the effects of the intensity and frequency of PA on EFs provide a comprehensive framework: in adolescence, for example, IC and CF improve with acute PA at moderate to high intensities, whereas WM responds more favourably to low or moderate intensities. This suggests a non-linear and domain-specific relationship between PA and EFs. Research on chronic interventions [150,156] shows that, particularly for WM, benefits require weeks to manifest, indicating a cumulative effect. This is especially relevant in educational settings, where medium- to long-term programmes are planned. However, to develop a more comprehensive and applicable understanding, longitudinal studies with longer follow-up periods, broader representation of various school ages, and standardised methodological protocols are necessary.

The most important finding of the present review is that the literature that simultaneously integrates sleep, PA, and EFs, although promising, is still very scarce. The few available studies suggest that sleep may mediate or moderate the effects of PA on EFs, and that PA, in turn, is capable of mitigating the negative effects of SD [158,159,160,161]. However, specific data are lacking for some age groups (for example, primary school) and for some executive components, such as CF, which is often overlooked in these combined studies. Furthermore, a comparison between the performance, quality, and duration of sleep between female and male students would be appropriate, especially for adolescents and young adults. It has been widely demonstrated that the menstrual cycle has a negative impact on the duration and quality of sleep and cognitive functions [166,167,168,169]. Given the absence of studies that also include the influence of the menstrual cycle in the relationship between PA, sleep, and EFs, future studies should therefore consider this factor. Furthermore, most studies in the literature consider the effect of sleep duration and quality on EFs without including the various components of sleep timing (e.g., wake-up, onset, midpoint) and within-person variability as additional variables. Future studies should examine these variables in parallel to obtain a clearer framework of the role of sleep in executive performance. The use of integrated experimental paradigms, which assess not only direct effects but also interactions between sleep and PA for EFs, could help to overcome the fragmentation of current evidence and in building more realistic models applicable to clinical and educational practices for students of different ages.

## 11. Conclusions

Healthy lifestyles, particularly sleep and PA, represent crucial modifiable factors for the development and strengthening of EFs and, consequently, for students’ academic performance. The evidence demonstrates that both sleep and PA significantly and often interdependently influence IC, WM, and CF. At a practical level, the findings suggest several educational and preventive strategies, such as the introduction of integrated programmes that combine moderate aerobic activity with cognitive stimulation, or the promotion of sleep education in the school and university contexts. Furthermore, the evidence supports the design of tailored interventions for age groups and specific executive domains, considering that the effectiveness of PA and sleep varies among children, adolescents, and young adults.

Despite the promising results, several gaps lead to some suggestions for future research:

1. An increased focus on children and adolescents: Research focuses mainly on university students; studies involving younger populations are essential for early interventions.
2. The standardisation of tools: The lack of validated tools for assessing SQ in younger individuals limits the comparability of results and the accuracy of interpretations.
3. The study of CF: This executive domain remains underexplored, particularly in studies combining sleep and PA. Given its relevance for academic adjustment, it is necessary to systematically include it in experimental protocols.
4. The development of integrated models: The few studies that simultaneously analyse sleep, PA, and EFs indicate a complex interaction among these variables. Integrated and longitudinal experimental paradigms able to measure direct, mediated, and moderated effects over time are essential for constructing ecologically valid models that are useful for educational and clinical practice.

**Figure 4:**
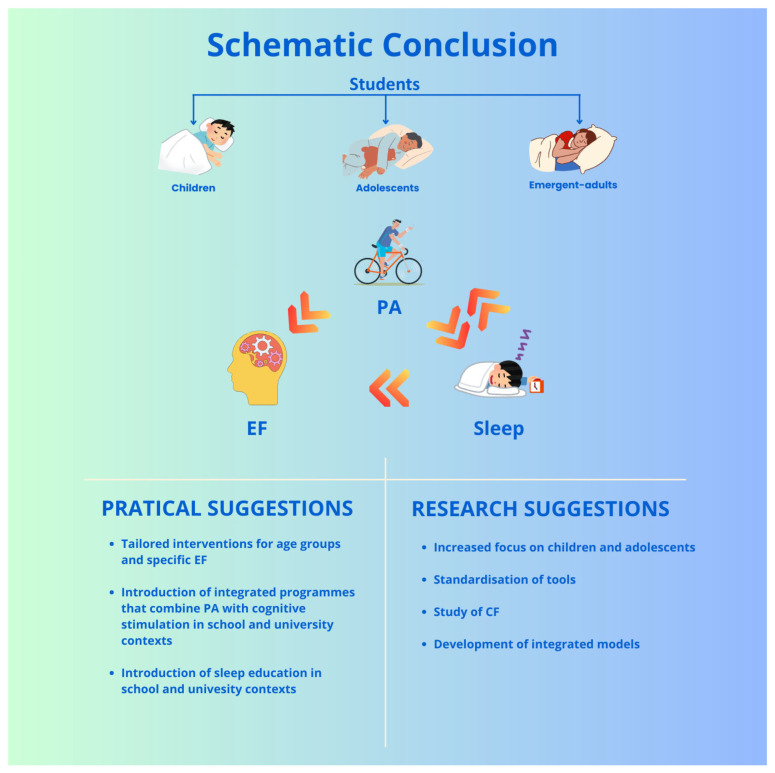
This figure summarises the conclusion of this narrative review, highlighting both practical and research aspect suggestions.

Figure 4 presents a schematic overview of the conclusions.

## Data Availability

No new data were created or analysed in this study. Data sharing is not applicable to this article.

## Supplementary Materials

The following supporting information can be downloaded at: https://www.mdpi.com/article/10.3390/clockssleep7030047/s1, Table S1: Main characteristics of the included studies. The table summarises the main characteristics of the studies included in the narrative review, such as sample size, gender ratio, mean age and standard deviation of the experimental and control groups, intervention, measures of EF, sleep and PA, and results.

## Abbreviations

The following abbreviations are used in this manuscript:

EF	Executive function
PA	Physical activity
WM	Working memory
IC	Inhibitory control
CF	Cognitive flexibility
dlPFC	Dorsolateral Prefrontal Cortex
vlPFC	Ventrolateral Prefrontal Cortex
mPFC	Medial Prefrontal Cortex
oFC	Orbitofrontal Cortex
FAT	Fronto-Aslant Tract
NREM	Non-rapid eye movement
REM	Rapid Eye Movement
SWS	Slow-wave sleep
SD	Sleep deprivation
TSD	Total sleep deprivation
ERP	Event-related potential
SJL	Social jet lag
IS	Insufficient sleep
SS	Sufficient sleep
SQ	Sleep quality
MET	Metabolic equivalent task
ATP	Adenosine triphosphate
HIIT	High-intensity interval training
OA	Obese adolescent
NWA	Normal-weight adolescent
