# Supplementary material for: Sleep, Physical Activity, and Executive Functions in Students: A Narrative Review
Source: Clocks Sleep. 2025 Sep 4;7(3):47. doi: 10.3390/clockssleep7030047 (PMC12452393; doi:10.3390/clockssleep7030047)
Supplement: Supplementary file 1 [file clockssleep-07-00047-s001.zip › clockssleep-3759954-supplementary.pdf]

Supplementary material

Table S1: Main characteristics of the included studies. The table summarises the main characteristics of the studies included in the narrative review, such as sample size, gender ratio, mean age and standard deviation of the experimental and control groups, intervention, measures of EF, sleep and PA, and results.

**Study**	**Experimental group** (N, % gender, mean age ± standard deviation)	**Control group** (N, % gender, mean age ± standard deviation)	**Intervention**	**Executive functions measure**	**Sleep measure**	**Physical activity measure**	**Results**
Warren, C. et al (2016).	377 elementary school students (female 47%)	332 students (female 47%)	No intervention	BRIEF-SR	Duration: self-report survey ad hoc created	Sedentary behavior: using three items adapted from the School-Based Nutrition Monitoring Student Questionnaire	Nightly sleep duration was significantly associated with sedentary behavior in elementary school students, and that this association was significantly mediated by children's EF.
D'Angiulli et al. (2023)	24 female high school students	No control group	No intervention	Inhibition: Stroop test and ERP analysis	Duration and quality: sleep	/	Reduced sleep duration in adolescents leads to impaired inhibitory

	(mean age=19,9 ± 0,4)				diary ad hoc created		control, as evidenced by both behavioural measures (more errors and slower reactions in Stroop tasks) and neurophysiological indices (less efficient ERPs, increased delta EEG activity due to fatigue).
Zhang et al. 2024	38 university students (50% female, mean age=20,46 ± 2.3)	34 university students (50% female, mean age=20,46± 2.3)	Acute SD (24 hours)	Cognitive flexibility: task switching, ERP analysis	/	/	Acute SD affected problem-solving effectiveness rather than efficiency, mainly because it systematically impaired cognitive processing associated with cognitive flexibility.
Cohen-Zion et al. 2017	190 high school students (59% female; mean age=16,3 ± 0,8)	No control group	No intervention	BRIEF-SR	Duration and quality: Modified School Sleep Habits Survey	/	The evening chronotype (preference for evening activities) and daytime sleepiness are predictors of deficits

							in daily executive functions and poorer academic performance in adolescents.
Pace-Schott et al. 2009	11 university students (age range 19-25; 100% male)	14 university students (age range 19-25; 100% male)	Total SD (35-39 hours)	Verbal fluency: Controlled Oral Word Association Test<br>Category fluency: Animal Naming Test<br>Logical reasoning: Baddeley Logical Reasoning<br>Problem solving: Tower of London<br>Working memory: Digit span backward; 2-Back<br>Decisional process:	Duration and quality: Nightcap, Stanford Sleepiness Scale	/	A single night of TSD did not cause significant deficits in EF performance compared to the control group

				Modified Iowa Gambling Task<br><br>Cognitive flexibility: Object Alternation<br><br>Inhibition: Haylings Sentence Completion Test; Stroop Color–Word Test<br><br>Olfactory discrimination: Smell Identification Test<br><br>Visual-spatial abilities: Mental Rotation Test (Shepard-Metzler)			
Yeung et al. 2018	20 university students (70% female; mean age= 19,6 ±	20 university students (80%	No intervention	Working memory: digit N-Back, near-infrared	/	/	Acute partial SD does not immediately impair working memory performance

	1,2) with partial SD	female; mean age= 20± 1,5) without partial SD		spectroscopy (NIRS)			in young adults, but it significantly reduces the activation of the prefrontal cortex involved in complex cognitive task processing.
Peng et al. 2020	16 university students (100% male, age range 21-28, mean age=23)	No control group	TSD (36 hours)	Working memory: 2-back and ERP analysis	/	/	A significant deterioration in WM performance was found. Through ERP analysis, the study also found a decrease in amplitude and an increase in latency of the N2 and P3 components. These alterations reflect a reduction in attentional resources and difficulty in suppressing responses and therefore in IC.
Cerolini et al. 2020	14 university students (21% male, mean age 23,21 ±	13 university students (23%	Cross-over study: each participant was assessed after a night of normal sleep and after a night of	Cognitive flexibility: Switch Cost Index	Sleep diaries (self-assessment)<br>Monitoring using a Zeo portable	/	Significant interaction Night × Group on the retroactive inhibition index: after SD, binge

	3,89) with binge eating behaviour.	male, mean age 24,69 ± 4,13)	partial SD (5 hours: going to bed after 1:00 a.m. and waking up before 6:00 a.m.); the order of the nights was counterbalanced.	Inhibition: Backward Inhibition Index	device to check sleep duration and times		eaters showed reduced inhibitory control compared to their usual night; no effect in controls. No significant difference between groups or conditions in the switch cost index.
Ballesio et al.	16 university students (56% female, mean age 23,31 ± 1,89) with Chronic Insomnia (CI)	16 university students (56% female, mean age 23,5 ± 2,19)	Each group was tested after a normal night's sleep and after a night of partial SD (5 hours allowed); order of nights counterbalanced.	Cognitive flexibility: Task Switching paradigm<br>Inhibition: Backward Inhibition	Sleep diaries (self-assessment)<br>Monitoring using a Zeo portable device to check sleep duration and times. Calculation of Total Sleep Time (TST, minutes) and Sleep Efficiency (SE, %)	/	Good sleepers: Switch Cost significantly reduced after deprivation, suggesting better performance in switching between tasks. Participants with insomnia: no difference between the two conditions. No significant effect on backward inhibition or number of correct responses. ICs (insomnia) showed lower sleep efficiency levels than GSs in both conditions and lower

							total sleep times. No significant differences or effects between conditions for the other measures.
De Angel et al. 2015	13 university students (61% female, mean age= 18,7 ± 2,2)	No control gorup	1. Baseline<br>2. Sleep reduction (only 4 hours of sleep last night)<br>3. Recovery sleep	Working memory: N-Back	/	/	Five days of sleep reduction compromises the phonological and visuospatial components of working memory, especially under conditions of high cognitive load. These effects manifest themselves in reduced accuracy and, for the visuospatial components, also in longer reaction times, indicative of greater cognitive effort required.
Jin et al. 2015	14 university students (100% male,	No control group	1. Baseline,<br>2. After 12 h of TSD,<br>3. After 24 h of TSD,	Inhibition: Go-No-Go task and EEG recordings	/	/	SD induced a dose-dependent functional decline in the

	mean age= 25,9, ± 2,3)		4. After 36 h of TSD,<br>5. Following 8 h of recovery sleep.				response inhibition of NoGo-N2 and NoGo-P3 on prefrontal cortex activation, and 8 h of RS resulted in recovery or mainte nance of the response inhibition. However, it was not restored to baseline levels.
Lau et al. 2015	40 university students (55% female, mean age=19,9) in nap-group	41 university students (55% female, mean age=19,9) in wake-group.	Nap (90 minutes) in the laboratory	Working memory: Psychomotor vigilance test, N-Back	Duration: PSG	/	A single monitored daytime nap lasting approximately 90 minutes improves working memory performance in university students; this benefit increases with longer sleep duration and, above all, with longer REM sleep during rest.
Higgins et al., 2020	120 elementary school (54,2% male, age range 6-10)	No control group	No intervention	Inhibition: Go/No-Go Task Working memory: Corsi Block Span	Pediatric Sleep Questionnaire and Daily Sleep Diary	/	Specific sleep parameters, particularly daytime sleepiness and sleep difficulties, have a significant impact on

				Set shifting: Trail-Making Test / Shape Trail Test Child Version (STT-CV)			both executive functions (particularly inhibition) and behavioural problems in school-aged children.
Anderson et al., 2009	62 “sleepy” middle and high school students (51,1% male, mean age= 13.7 ± 0.8)	174 “non-sleepy” middle and high school students (52% male, mean age= 13 ± 0.8 anni)	No intervention	BRIEF<br>D-KEFS (Delis-Kaplan Executive Functioning System)	Epworth Sleepiness Scale;<br>Wrist actigraphy<br>Sleep diary;<br>PSG	/	There is a high prevalence of self-reported sleepiness in adolescents and that this sleepiness is significantly linked to modest deficits in executive functions.
Ouellet et al. 2024	3517 middle school students (49.5% female, mean age= 12.8 ± 0.45 anni,)	No control group	No intervention	Inhibition: Passive Avoidance Learning Paradigm<br>Working memory: Find the Phone Task	Self-reported sleep questionnaire ad hoc created	/	Low sleep quality is associated with poorer executive performance and is strongly linked to inhibitory control.

Gong et al. 2024	30 university students (55% female, mean age 19,23 ± 1,05) with low sleep quality	30 university students (55% female, mean age 19,23 ± 1,05) with high sleep quality	No intervention	Working memory: color recall task	PSQI	/	Students with high sleep quality retained significantly more information than those with low sleep quality.
Parrilla et al. 2024	18 university students (mean age 29,86) with low sleep quality (≥5 PSQI)	11universi ty students (mean age 29,86) with high sleep quality (<5 PSQI)	No intervention	BRIEF<br>Frontal Systems Behaviour Scale	PSQI	/	Individuals in the poor sleep quality group were reported as having significantly worse executive function and working memory scores. Young adult college students who report less than 7 hours of sleep per night have lower scores on informant measures of working memory and executive function.
Almarzouki et al., 2022	83 university students	No control group	No intervention	Working memory:	PSQI	/	Poorer sleep quality (disturbances,

	(42,2% male, mean age= 21,8 ± 1,59)			Cambridge Neuropsychological Test Automated Battery, spatial working memory item.			duration, use of sleep medication) was correlated with less effective working memory performance.
Conner et al., 2015	284 university students (49,4% male, mean age= 9,81 ± 3,83)	No control group	No intervention	Barkley Deficits in Executive Functioning Scale-Long Form	PSQI		Among university students, there is a statistically significant relationship between sleep quality, alcohol consumption and executive functioning. In particular, poorer sleep quality was found to be associated with greater difficulties in executive functions (e.g. time management, organisation, self-control, motivation and emotional regulation). Furthermore, alcohol consumption acts as a partial mediator in this relationship: students with sleep

							problems tend to engage in riskier alcohol use, which in turn exacerbates executive dysfunction.
Abbas et al., 2020	17 university students (76% female, age range 20-25)	No control group	Sleep hygiene protocol: room temperature 18-20°C, no electronic devices before bedtime, use of blue light lenses if necessary, no caffeine after midday, daily exposure to natural light, only warm light in the evening, regular sleep schedule (bedtime 10-11 p.m., wake up at dawn), no physical activity close to bedtime.	Inhibition: Gradual Onset Continuous Performance Test	OURA ring (sleep index: sleep quality, duration, efficiency, REM, deep, light, latency, resting HR); subjective sleep diary	Physical activity recorded in the sleep diary if longer than 20 minutes; no physical activity allowed close to bedtime	The experimental group reported more positive moods; sleep quality was positively (but not significantly) correlated with performance on the inhibition test; REM alone was significantly associated with better performance on response inhibition tasks.
Chen et al., 2024	55 university students (56,9% female, mean age= 20,06 ± 0,92) with insomnia (ISI >7; PSQI >5)	47 university students (56,9% female, mean age= 20,06 ± 0,92) without	No intervention	Cognitive flexibility: Cognitive Flexibility Inventory (CFI); Number-Letter Task (N-L task), ERP analysis.	PSQI; Insomnia Severity Index (ISI) used as exclusion/inclusio n criteria for the experimental group and the control group	/	Students with insomnia show lower CFI scores, higher switch costs, longer reaction times, lower accuracy, and alterations in brain ERP components (P2, N2, P3) compared to

		insomnia (ISI ≤7; PSQI ≤4)					the control group. Insomnia is associated with a reduction in cognitive flexibility potentially mediated by neurophysiological changes.
Ren et al., 2024	90 right-handed elementary students (48.9% females; age range 7-8)	20 right-handed elementary students (48.9% females; age range 7-8)	CHRONIC PA<br>Three groups:<br>● PA (physical activity): 23 children;<br>● IA (intellectual activity): 25 children;<br>● PA+IA: 22 children.<br>All three received 40 minutes/day, 4 days/week for 16 weeks of moderate physical exercise (e.g. jumping rope, sports games) or/and cognitive activities (drawing, chess, programming).	Inhibition: Animal Go/No-Go<br>Working memory: Working Memory Span Task<br>Reaction time: Simple Reaction Test<br>Cognitive flexibility: Flexible Item Selection Task	/	- Cardiovascular fitness: 50 m run<br>- Muscle strength: handgrip strength, standing long jump<br>- Speed sensitivity: 4 × 10 m shuttle run<br>- Flexibility: sit and reach	16 weeks of structured aerobic activity, performed regularly (4 days a week, 40 minutes per session), has a very positive impact on both physical fitness and executive functions in primary school children. The improvements were more pronounced in the group that performed both physical and intellectual activities simultaneously (PA+IA), suggesting a synergistic effect of

							the multidimensional approach.
Erwin at al., 2024	51 middle school students (62% male; age range 13-14)	No control group	Acute PA (1 session)<br>- Aerobic: running tag game (continuous running game)<br>- Anaerobic: interval dumbbell workout (interval training with dumbbells)	Inhibition: Go/No Go task, Flanker test;<br>Working memory: Stroop color-word task<br>Reaction time	/	/	Aerobic: Significantly improves both reaction time and the number of correct responses related to WM.<br>- Anaerobic: Improves only reaction time of WM.<br>- No significant effect in the Flanker and Go/No Go tests for any of the conditions.
Hu et al., 2024	28 obese middle school students (53,6% female)	24 normal-weight middle school (53,6% female)	Combined exercise: 4 times/week, 40 minutes/session (5 min warm-up, 20 min aerobic exercise, 10 min resistance, 5 min cool-down), moderate intensity (65% HR max) for 14 weeks. Mainly aerobic exercises, supplemented with	Inhibition: Flanker task<br>Working memory: 1-back task<br>Cognitive flexibility: More-odd shifting task	/	/	Obese: EF initially lower in the domains of inhibition and cognitive flexibility. After intervention:<br>- Inhibition and cognitive flexibility improve significantly at 12 and 14 weeks.<br>- Working memory improves

			bodyweight resistance exercises.	Assessed at pre-test, after 12 and 14 weeks			significantly at 14 weeks.<br>- No significant improvement in EF in the control group.
Ludyga et al., 2018	51 university students (58,8 % female; mean age= 21,8 ± 1,3)	No control group	A single session of moderate aerobic exercise (20 minutes of running, 70% HRmax) with warm-up (3 minutes) and cool-down (2 minutes)	Inhibition: Flanker task, reaction time and accuracy;<br>Working memory: n-back task, reaction time and adjusted hit rate	/	IPAQ: used for inclusion/exclusion criteria. Only subjects with >3 days of vigorous activity or >7 days of mixed activity >3,000 MET min/week were included.	A single session of moderate aerobic exercise (20 minutes of running at 70% of maximum HR) provides selective benefits to cognitive function in university students. In particular, acute physical exercise improved inhibitory control (the ability to inhibit automatic responses and manage distractions), as well as short- and long-term verbal memory, compared to a sedentary control condition (reading a text). No significant differences were

							found in working memory.
Li et al., 2014	15 university students (100% female, mean age= 19,56 ± 0,81)	No control group	Control condition: 20-minute sitting rest session (no physical activity)<br>Acute aerobic exercise session on a cycle ergometer: 5 min warm-up, 20 min at moderate intensity (120 bpm, 60-70% max HR), 5 min cool-down	Working memory: N-back, reaction time during fMRI	/	/	No significant behavioural improvement in working memory (accuracy/reaction time). Acute exercise significantly altered brain activation (increase in the right middle frontal gyrus, right lingual gyrus and left fusiform gyrus; decrease in the anterior cingulate cortex, left inferior frontal gyrus, right paracentral lobule) under high cognitive load.
Martínez-Díaz et al., 2020	25 physical education university students (100% male, mean age 21,7 ± 2,1)	No control group	HIIT: 10 × 1 minute of cycling at VO2 peak (power at maximum VO2), with 1 minute of passive recovery between each interval. Cadence: 70 rpm. Sessions carried out	Working memory: Digit Span Test forward, backward and total (DST)	/	Graded exercise test on a cycle ergometer to determine VO2 peak and test body composition	Improved working memory: increased DST scores, peaking immediately after HIIT and still higher than baseline values after 30 minutes

			between 8:00 and 10:00 in the morning.			(impedance measurement). During HIIT: HR monitoring, measurement RPE using the Borg Scale 6–20.	
Liu et al., 2024	38 elementary school children (60% male; age range 9-10)	20 elementary school children (60% male; age range 9-10)	Three groups:<br>- HIIT (N=19): High-intensity interval training (≥85% HRmax, 1 min jumping + 1 min rest x 5 sets)<br>- MICT (N=19): Moderate-intensity continuous training (60-80% HRmax, 5 min continuous jumping)<br>- CON (N=20): Resting in place, no activity	Inhibition: Stroop Color-Word Test;<br>Working memory: Digit Span Test-Backward<br>Alert, executive control and orientation: Attention Network Test	/	Heart frequency monitored in real time with Polar Verity Sense	-HIIT and MICT: improve working memory, executive control network efficiency, and response speed in inhibitory control; no significant change in accuracy.<br>- HIIT alone: also improves attention orienting network efficiency.<br>- No significant difference between HIIT and MICT in executive function; no benefit observed in the control group.

Peruyero et al., 2017	44 high school students (52,3% male, mean age= 16,39±0,68)	No control group	Two conditions:<br>1) 20-minute Zumba session at low-moderate intensity<br>2) 20-minute Zumba session at moderate-vigorous intensity<br>(both with a 5-minute warm-up and cool-down)<br>All participants performed all conditions, assigned in rotation to counterbalance the order.	Inhibition: Stroop Test	/	Intensity monitored using ActiGraph GT3X accelerometers; duration of activities divided into light, moderate and vigorous intensity according to age-specific thresholds; percentage of time spent at various intensities analysed.	- Moderate-vigorous exercise significantly improved inhibitory control compared to both no exercise and light-moderate exercise ($p < 0.001$, high effect size)<br>- Mild-moderate intensity sessions: small improvements, not significant compared to control<br>- Vigorous exercise alone has marked benefits on inhibitory executive functions
Budde et al., 2009	Group 1: 18 high school students (50% male, mean age 14,38 ± 0,50)<br>Group 2: 20 high school students (55%	21 high school students (61% male, mean age= 14,50±0,5)	Two experimental groups:<br>- Group 1: low-intensity running (50–65% of maximum HR) for 12 minutes<br>- Group 2: high-intensity running (70–85% of	Working memory: Letter Digit Span	/	Measured in minutes per week using questions	-Low-intensity acute exercise significantly improved working memory in low performers.<br>- High-intensity exercise increased testosterone and cortisol but did not

	male, mean age 14,24 ± 0,56)		maximum HR) for 12 minutes<br>Intensity was adjusted individually by monitoring HR<br>Control group: no physical activity; students seated in class for 12 minutes during the intervention				improve working memory.<br>- No link between cortisol change and executive performance; high post-exercise testosterone correlated with (non-significant) worsening of working memory.
Berse et al., 2015	227 high school students (53% male, mean age =14,8 ± 0,9)	No control group	2 conditions<br>1. Viewing an episode of an educational cartoon, sitting in a relaxed position. Duration comparable to the experimental condition (approximately 10–14 minutes)<br>2. Acute, intense physical exercise: two intervals on a cycle ergometer (increase of 25 watts every 10 seconds, target speed of 70 RPM), until	Cognitive flexibility: switching task	/	Physical activity habits: 5-point self-assessment scale<br>Fitness test: incremental running on a track, measurement of anaerobic threshold using lactate and HR	Acute, intense physical exercise significantly reduced switch costs (improvement in shifting). Effect predicted by dopaminergic polymorphisms.

			exhaustion, with lactate and HR monitoring<br><br>Each participant performed both the experimental and control conditions.				
Robinson et al., 2022	1.Sedentary with cognitive training (SECT): 21 high school students (62% female, mean age 15,65 ± 0,49)<br><br>2. Resistance training without cognitive training (RTNC): 29 high school students (59 % female, mean age 15,9 ± 0,41)	23 high school students (65% male, mean age=15,78 ± 0,42)	- Control: sedentary condition without cognitive training: administrative activities in class (roll call, homework), no formal activities or exercise for 6-8 minutes 3 4<br>- Sedentary with cognitive training (SECT): cognitive training only, no exercise<br>- Resistance training no cognitive (RTNC): bodyweight resistance exercises without cognitive component<br>- Resistance and cognitive training (RTCT): bodyweight resistance exercises with	Inhibition: Flanker Inhibitory Control and Attention Test<br>Cognitive flexibility: Dimensional Change Card Sort Test<br>Episodic memory: Picture Sequence Memory Test	/	- Acute physical activity measured using the Borg RPE (CR10 scale) on a smartphone app (before and after each session)<br><br>- Muscle fitness measurements: 90° push-up test, 30-second squat-to-chair test, plank hold test (assessing upper/lower body and core endurance)	No significant effect on cognitive inhibition or flexibility by any exercise group, compared to the control group<br><br>- Significant improvement in episodic memory only in the RTNC group (resistance training without cognitive component)<br><br>- No improvement in muscular fitness in the exercise groups; sedentary groups

	3.Resistance + cognitive training (RTCT): 24 high school students (75% male, mean age 15,8 ± 0,40)		simultaneous cognitive training<br>12 sessions in total, 3 per week x 4 weeks, approximately 6-8 minutes each				sometimes performed better in muscle tests<br>- All groups (except the control group) improved students' on-task behaviour in class<br>- Exercise intensity classified as “light” to 'moderate'
Rao et al., 2019	411 adolescent students	391 adolescent students	Experimental group performed structured yoga (n = 411) for 1 hour per day for 2 months<br>Control group performed structured exercise (n = 391) for 1 hour per day for 2 months	Cognitive flexibility: Trail Making Test number version and alphabetic version (TMTN, TMTA)	/	/	Both groups (yoga and exercise) improved significantly in TMTN. The yoga group showed a significantly greater improvement in TMTA than the exercise group. No significant difference between groups in TMTN.

Jeon et al.,2017	30 middle school students (100% male, mean age= 15,22 ± 0,61)	10 middle school students (100% male, mean age= 5,05 ± 0,41)	Aerobics on a treadmill 4 times/week for 12 weeks<br>LIEG: Low Intensity Exercise Group – Group performing low-intensity aerobic exercise (40% of oxygen reserve, VO2R)<br>MIEG: Moderate Intensity Exercise Group – Group performing moderate-intensity aerobic exercise (55% VO2R)<br>HIEG: High Intensity Exercise Group – Group performing high-intensity aerobic exercise (70% VO2R)<br>SG: Stretching Group – Control group performing only stretching exercises for 30 minutes, 4 times/week for 12 weeks	Working memory: numerical subtest of the Wechsler Scale for Children K-WISC-III	/	VO2max via graded exercise testing (GXT); calculation of target VO2 and monitoring of HR and energy expenditure for each session. Monitoring via treadmill with specific calculation of duration for 200 kcal.	Moderate- and especially high-intensity aerobic exercise has positive effects on resting levels of BDNF (brain-derived neurotrophic factor) and cognitive functions, particularly working memory, in adolescents whose brains are still developing. In particular, only the group that performed high-intensity exercise (HIEG) showed a significant improvement in working memory, while the moderate-intensity group showed an increase in BDNF levels without, however, achieving concrete improvements in executive functions.

							The low-intensity and stretching groups did not report any significant benefits.
Aguirre-Loaiza et al. (2022)	9 university students (78% male, mean= age 23,3 ± 1,6)	10 university students (80% male, mean age=23,3 ± 1,6)	Experimental group: 20 minutes of high-intensity cycling (80-90% HRmax, on a cycle ergometer), with a 5-minute warm-up and 5-minute cool-down; direct supervision of intensity via HR monitor; subjects exercise individually.<br><br>Control group: Sedentary waiting in the same room for 20 minutes.	Inhibition: Stroop Test	/	HR monitored	Shorter reaction times after exercise compared to the control group; accuracy tended to remain stable or decrease slightly after exercise (more errors after cycling).
Wen et al., 2023	47 university students (59,5% male, mean age= 19,17 ± 1,94)	No control group	Slackline: 50 minutes total (5 minutes warm-up + 15 minutes familiarisation/instruction + 30 minutes active exercise on slackline); activity involves movement on suspended webbing; increasing difficulty; bodyweight exercise; indoor	Inhibition and cognitive flexibility: Modified Simon Task	/	RPE questionnaire	Reduction in reaction times in inhibition after slacklining, with no changes in accuracy; selective effectiveness on inhibition in high-demand tasks.

			environment; HR and RPE monitoring.<br>Baseline: Viewing of a neutral film of the same duration in a similar environment (sedentary).				
Yang et al., 2024	25 university students (100% male; mean age= 20,20 ± 0,91)	No control group	4 conditions (all on cycle ergometer, 20 min)<br>1) Rest (control condition)<br>2) Moderate exercise (40-60% HRR)<br>3) Moderate exercise + cognitive demand (40-60% HRR + Flanker during)<br>4) Moderate exercise + high intensity (60-90% HRR)<br>Adjusted intensity, randomisation control, effort assessment.	Inhibition: Flanker Task	/	HR monitored, RPE questionnaire, NASA- *Task Load Index*	All exercise conditions improve inhibition (RT); greater effect with high intensity and high cognitive load; accuracy does not vary; HI greater fatigue.
Fan et al.,<br>2021	15 university students (60% male, mean age= 20.20 ±	No control group	Cycle ergometer cycling, 30 minutes total (15 minutes central phase) at 3 intensities (low: 35-50%,	Inhibition: Go-No-Go; Franker test	/	HR monitored, RPE questionnaire	Dose-dependent improvement in inhibition (Go/NoGo), with maximum

	0.94);<br><br>Low, medium and high intensity: 5 subjects/group x 3 groups		moderate: 50-70%, high: 70-85% HRmax).				benefit at moderate intensity; no effect on interference (Flanker).zzz
Martínez-Díaz et al., 2023	25 university students (100% male, mean age= 21,7 ±2,1)	No control group	Single HIIT session on a cycle ergometer:<br>- 10 intervals of 1 minute each at the load corresponding to individual VO2peak<br>- 1 minute of passive rest between each interval<br>- Constant HR monitoring<br>- RPE assessed at the end of each interval using the Borg scale 6–20<br>- Mean arterial pressure measured halfway through the session	Working memory: Digit Span Test (WAIS IV) forward (DST-D), backward (DST-I) and total (DST-T)	/	Level of physical activity measured using IPAQ: 5877.6 ± 1668.2 MET/min/week; VO2peak: 47.1 ± 9.3 mL/kg/min	After HIIT, acute improvement in working memory (increase in DST-T and DST-I).
Ji et al., 2017	20 university students	No control group	Single treadmill running session, moderate	Elaboration velocity:	Not measured directly. Used as	Level of physical activity	- Moderate acute exercise improves

	(100% male, mean age= 23,5 ± 2,0)		intensity (60% of heart rate reserve - HRR), 30 minutes; same protocol in both temperate (25°C) and cold (10°C) environments.	Identification task of Battery CogState<br>Working memory: 2- Back of Battery CogState<br>Cognitive flexibility: Set-shifting of Battery CogState<br>Reaction times and accuracy were measured..	an exclusion criterion: participants with less than 12 hours of sleep in the 48 hours prior to the test are excluded.	assessed using IPAQ; all participants classified as "moderate physical activity" (600–3000 MET minutes/week). Exercise intensity in the laboratory monitored using a HR monitor (Polar H7) and %HRR.	processing speed and working memory (but not cognitive flexibility) in both temperate and cold environments, but in cold environments the effect is not maintained after 30 minutes.<br>- No effect detected on cognitive flexibility.<br>- Benefits on executive functions associated with increased skin temperature and thermal sensation after exercise.
a) Wang et al., 2023	28 university students (43% male, mean age=20,17±1,6 6)	27 university students (48% male, mean	Experimental group: 12 weeks, 3 times a week, Yang-style Tai Chi (24 forms), 60 minutes per session + 30 minutes/day of independent practice required; moderate intensity (57-69% HRmax)	Working memory: Visual 2-back task	/	Intensity monitoring (HR, Polar RS800CXSD); there are no direct measurements of motor fitness	Tai Chi significantly improved performance in the 2-back task (greater accuracy, lower response times).

		age=19,93± 1,74)	monitored using a HR monitor<br>Control group: performed traditional non-cognitive sports exercises at the same intensity (monitored with a HR monitor) but without Tai Chi.			in the main results, only verification that the intensity was comparable between groups.	
b) Wang et al., 2024	35 university students (29% male, mean age= 20,08±0,28)	35 university students (17% male, mean age= 20,71±0,51)	Experimental group: 12 weeks, 3 times a week, 24-form Tai Chi, 45 minutes per session (5 minutes warm-up, 35 minutes practice, 5 minutes relaxation), supervised by instructors, HR monitoring (~55% HRmax)<br>Control group: maintained their normal daily routine, without any new physical activities	Inhibition: Stroop Color-Word Test, Pre/post-surgery frontal EEG (θ, α, β band power) as a functional index of inhibitory/executive control	/	Intensity monitoring (sports wristband for HR); no direct physical measurements reported in the main results	Tai Chi practitioners showed a significant increase in accuracy and a reduction in time in the incongruent Stroop (inhibitory control), as well as an increase in frontal θ and α powers in the post-intervention EEG; no significant changes were observed in the control group.
Tavakoli et al., 2024	30 middle school students (100% male, mean	30 middle school students (100% male,	Experimental group: 8 weeks, 3 sessions/week, each lasting 45 minutes, multi-joint exercises for major muscle groups	Inhibition: Simon Task<br>Working memory: N-back	Adolescent Sleep Wake Scale	/	Significant improvement in sleep quality (d=1.61 post, 0.92 follow-up). Greater increases vs

	age=12,13 ± 0,28)	mean age=12,83 ± 0,61)	(shoulders, arms, hips, legs), 3 sets of 8-12 repetitions, intensity 60-75% 1RM.<br><br>Control group: They participate in two meetings per week (small groups), each lasting 45 minutes, for 8 weeks 1. During these meetings, they engage in social and recreational activities.	Cognitive flexibility: Wisconsin Card Sorting			control on inhibitory control (d=1.23 post, 0.60 follow-up), working memory (d=1.93 post, 1.32 follow-up), cognitive flexibility (d=2.06 post, 1.63 follow-up). Effects persisted at 4 weeks.
Sun et al., 2022	212 elementary and middle school students (50% male, mean age= 12,47 ± 1,76)	No control group	No intervention	Inhibition: Eriksen Flanker Task<br><br>Working memory: Sternberg Working Memory Task	Cleveland Adolescent Sleepiness Questionnaire	Actigraph GT3X accelerometer, worn for 7 days, data: % sedentary time, % Low-PA, % Moderate/Vigorous-PA; objective data on activity intensity and duration	Low PA and moderate/vigorous PA correlate positively with working memory performance by reducing sleepiness at school. Therefore, increased sedentary behaviour leads to increased sleepiness and, in turn, poorer working memory. No mediation on the measure of inhibitory control.

Li et al., 2021	180 university students (39% male, mean age= 20,15 ± 1,92)	No control group	No intervention. Division into groups by PA level (IPAQ: low/medium/high)	Inhibition: Stroop Test	PSQI	IPAQ	Sleep efficiency and subjective sleep quality are mediators between physical activity and executive functions (in particular inhibitory control): greater PA is linked to better inhibitory control through better sleep.
Liu et al., 2022	30 university students (50% male, mean age= 22,3 ± 1,3)	No control group	Each participant performs both a control condition (30 minutes of seated rest after a night of TSD) and an intervention (30 minutes of moderate aerobic exercise on a stationary bike, 60-69% of maximum heart rate, immediately after TSD). Randomised order, minimum distance between the two tests. Functions measured before and after each condition.	Inhibition: Go/NoGo Task	PSG	/	A sleepless night impairs inhibitory control.<br>Acute exercise improves performance and inhibition immediately, 30 minutes and 1 hour after exercise compared to control (rest). Temporary improvement suggests acute effect of physical activity.

Legend: BRIEF-SR: Behavioral Rating Inventory of Executive Function-Self-Report; SD: sleep deprivation; TSD: total sleep deprivation; REM: rapid eye movements; ERP: Event Related Potential; EEG: Electroencephalography; PSQI: Pittsburgh sleep quality index; IPAQ: International Physical Activity Questionnaire; fMRI: Functional magnetic resonance imaging; HIIT: High-Intensity Interval Training; HR: heart rate; HRR: heart rate reserve; RPE: Rating of Perceived Exertion; MET: metabolic equivalent of task.
